# Development and validation of a predictive model for submucosal fibrosis in patients with early gastric cancer undergoing endoscopic submucosal dissection: experience from a large tertiary center

**DOI:** 10.1080/07853890.2024.2391536

**Published:** 2024-08-16

**Authors:** Yunqing Zeng, Jinhou Li, Yuan Zheng, Di Zhang, Ning Zhong, Xiuli Zuo, Yanqing Li, Wenbin Yu, Jiaoyang Lu

**Affiliations:** a Department of Gastroenterology, Qilu Hospital of Shandong University, Jinan, Shandong, China; b Department of Gastroenterology, Taian City Central Hospital, Taian, Shandong, China; c Department of Medical Oncology, Qilu Hospital of Shandong University, Jinan, Shandong, China; d Department of General Surgery, Qilu Hospital of Shandong University, Jinan, Shandong, China

**Keywords:** Early gastric cancer, endoscopic submucosal dissection, predictive model, severe fibrosis, submucosal fibrosis

## Abstract

**Background:**

Submucosal fibrosis is associated with adverse events of endoscopic submucosal dissection (ESD). The present study mainly aimed to establish a predictive model for submucosal fibrosis in patients with early gastric cancer (EGC) undergoing ESD.

**Methods:**

Eligible patients with EGC, identified at Qilu Hospital of Shandong University from April 2013 to December 2023, were retrospectively included and randomly split into a training set and a validation set in a 7:3 ratio. Logistic regression analyses were used to pinpoint the risk factors for submucosal fibrosis. A nomogram was developed and confirmed using receiver operating characteristic (ROC) curves, calibration plots, Hosmer-Lemeshow (H-L) tests, and decision curve analysis (DCA) curves. Besides, a predictive model for severe submucosal fibrosis was further conducted and tested.

**Results:**

A total of 516 cases in the training group and 220 cases in the validation group were recruited. The nomogram for submucosal fibrosis contained the following items: tumour location (long axis), tumour location (short axis), ulceration, and biopsy pathology. ROC curves showed high efficiency with an area under the ROC of 0.819 in the training group, and 0.812 in the validation group. Calibration curves and H-L tests indicated good consistency. DCA proved the nomogram to be clinically beneficial. Furthermore, the four items were also applicable for a nomogram predicting severe fibrosis, and the model performed well.

**Conclusion:**

The predictive models, initially constructed in this study, were validated as convenient and feasible for endoscopists to predict submucosal fibrosis and severe fibrosis in patients with EGC undergoing ESD.

## 1. Introduction

Early gastric cancer (EGC) is defined as gastric cancer confined to the mucosa or submucosa (T1 cancer), irrespective of lymph node metastases [1]. Endoscopic submucosal dissection (ESD) is a technically demanding procedure and has become a standard treatment for EGC [2]. Submucosal fibrosis beneath the lesions can contribute to the technical difficulties of what is already a very technically demanding technique [3]. Submucosal fibrosis is associated with prolonged operative time, increased risk of complications such as perforations, and reduced complete en bloc resection rate [4–6]. Severe submucosal fibrosis complicates the identification and separation of the appropriate submucosal layer from the muscular layer. This difficulty in recognizing the proper dissection line and submucosal vessels can lead to erroneous incisions and challenges in hemostasis [7–10]. Hence, the mean ESD procedure time, complete en bloc resection rate, and overall perforation rate further increase significantly in severe fibrosis [2,11]. In clinical practice, compared with lesions with mild fibrosis, gastric ESD for lesions with severe fibrosis requires highly advanced dissection techniques and a high degree of expertise and precision [12]. Therefore, identifying the grade of fibrosis is also important for the formulation of ESD strategy.

Considering that submucosal fibrosis can only be detected intraoperatively, an adequate pre-procedural prediction for EGC with submucosal fibrosis is vital for an accurate stratification of ESD complexity and the estimation of procedure duration, thereby allowing the planning of a proper endoscopic strategy or even referral to a high-volume center [13]. To date, no study has been specifically designed to predict the probability of submucosal fibrosis in patients with EGC before ESD.

Herein, we identified the predictive factors and developed a model to more accurately assess the risk of submucosal fibrosis preoperatively and provide a practical tool for physicians to predict submucosal fibrosis in EGC for clinical decision-making. In parallel, we also performed a similar analysis for EGC with severe submucosal fibrosis.

## 2. Materials and methods

### 2.1. Patients

We retrospectively retrieved the medical records of patients with early gastric adenocarcinoma undergoing ESD at Qilu Hospital of Shandong University between April 2013 and December 2023. The diagnosis of early gastric adenocarcinoma was based on post-ESD pathology reports. The main exclusion criteria were as follows: lesions recurring after endoscopic or surgical resection; inadequate endoscopic information for determining the grade of submucosal fibrosis; and patients with missing endoscopic forceps biopsy (EFB) results. The following clinicopathologic characteristics were selected and involved in our study: the sex and age of the patient; use of antithrombotics; comorbidity; biopsy history; biopsy result; lesion size and location; and various endoscopic findings of EGCs including depression, redness, atrophy, dyke-like elevated margins, white fur, spontaneous bleeding, and ulceration. The determination of ulceration in this study included open ulcer and ulcer scar. Approval for the study protocol was granted by the Medical Ethics Committee of Qilu Hospital of Shandong University (KYLL-202404-049). The need for obtaining informed consent was exempted due to the retrospective nature of this study.

### 2.2. ESD procedure

The indication for ESD of EGC was established based on the criteria of the Japanese Gastric Cancer Association [14]. All ESD procedures were conducted by experienced ESD endoscopists using a conventional single-channel upper gastrointestinal endoscope (EG29-i10, Pentax Medical, Shanghai, China; GIF-260J, Olympus, Tokyo, Japan; or GIF-450RD5, FUJIFILM Medical, Tokyo, Japan). The lesion was lifted by submucosal injection of saline solution and indigo carmine mixture. A Dual knife (KD-650L, Olympus), FLUSH knife (DK2618JB, FUJIFILM), or IT knife (KD-611L, Olympus) was used for a circumferential mucosal incision and submucosal dissection. Finally, the resected tissue was extracted by suction.

### 2.3. Definitions

**Figure 1.**
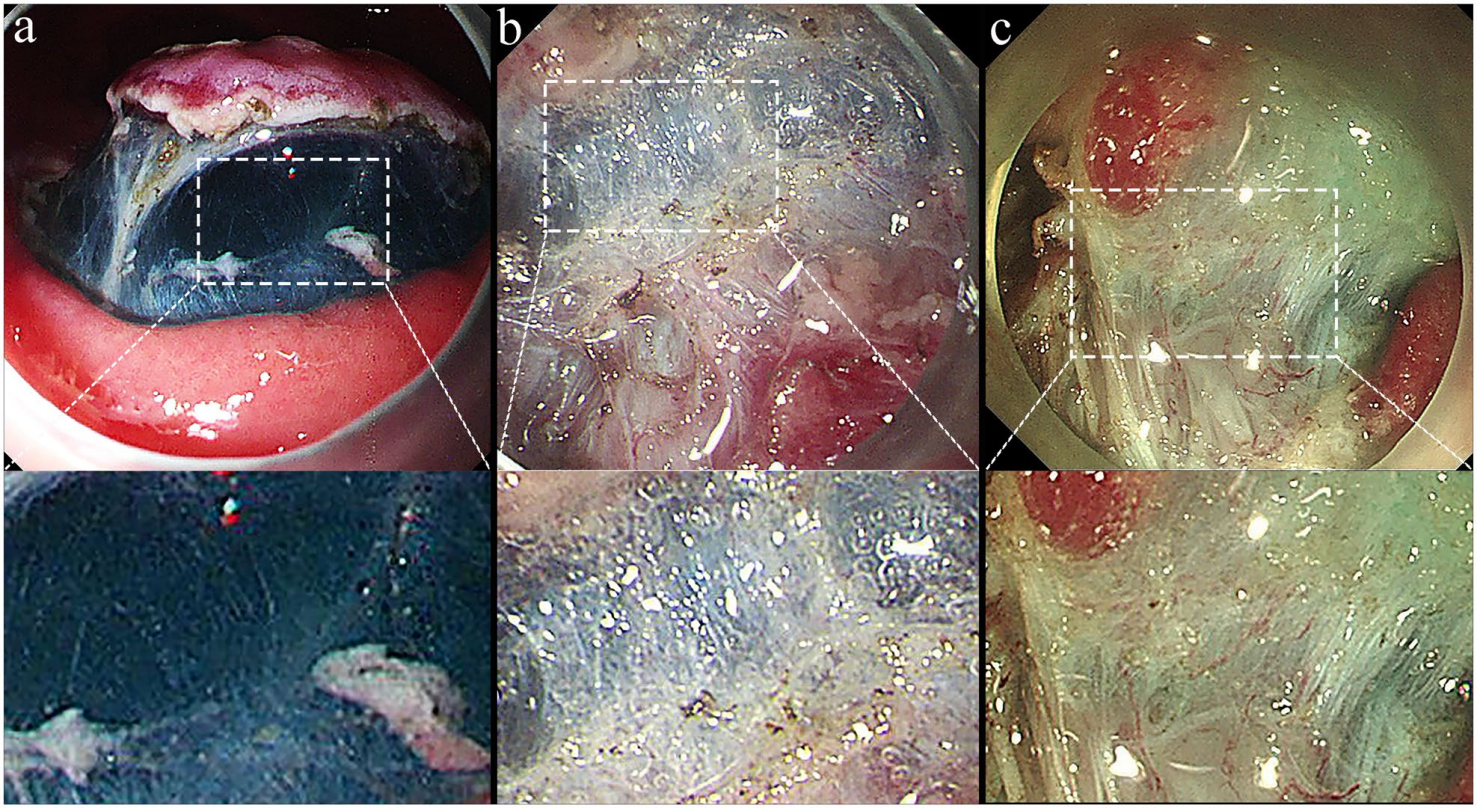
Degree of endoscopic submucosal fibrosis in early gastric cancer. (a) F0, no fibrosis, presenting as a blue transparent layer. (b) F1, mild fibrosis, appearing as a white web-like structure in the submucosal layer. (c) F2, severe fibrosis, manifesting as a white muscular structure without a transparent layer in the submucosal layer.

The degree of submucosal fibrosis was classified into three grades based on the findings of the submucosal layer at the time of injection of indigo carmine solution: F0 (no fibrosis), presenting as a blue transparent layer (Figure 1a); F1 (mild fibrosis), appearing as a white web-like structure in the submucosal layer (Figure 1b); and F2 (severe fibrosis), manifesting as a white muscular structure without a transparent layer in the submucosal layer (Figure 1c) [2,11,15,16]. The extent of fibrosis was evaluated independently by two endoscopic physicians and any discrepancies were resolved by consensus discussion with endoscopists who performed the ESD.

### 2.4. Statistical analysis

Although some of the patients had multiple neoplasms treated by ESD, quantities observed in various procedures were assumed to constitute statistically independent observations for analytical purposes. Cases were randomly divided into a training set and a validation set in a 7:3 ratio. Normally distributed quantitative variables were expressed as mean ± standard deviation and compared using the Student’s t-test. Qualitative variables were expressed as percentages and compared using the Chi-square (χ^2^) or Fisher’s exact tests. Univariable logistic regression analysis was performed to identify factors associated with submucosal fibrosis or severe fibrosis during ESD in the training group. Only variables with *p* < 0.05 in the univariate analysis were included in the multivariable analysis. Then, multivariate analysis was used to determine independent influencing factors, which were utilized to construct a nomogram prediction model. The discriminatory capacity of the model was determined by the area under the receiver operator characteristic (ROC) curve (AUC). Calibration of the nomogram was scrutinized through a calibration curve and the Hosmer-Lemeshow (H-L) test (*p* > 0.05 indicates favourable agreement). Decision curve analysis (DCA) was employed to evaluate the net benefit ratio of the model across various threshold probabilities. All the statistical analyses were performed using R software 4.3.0.

## 3. Results

### 3.1. Baseline characteristics

**Figure 2.**
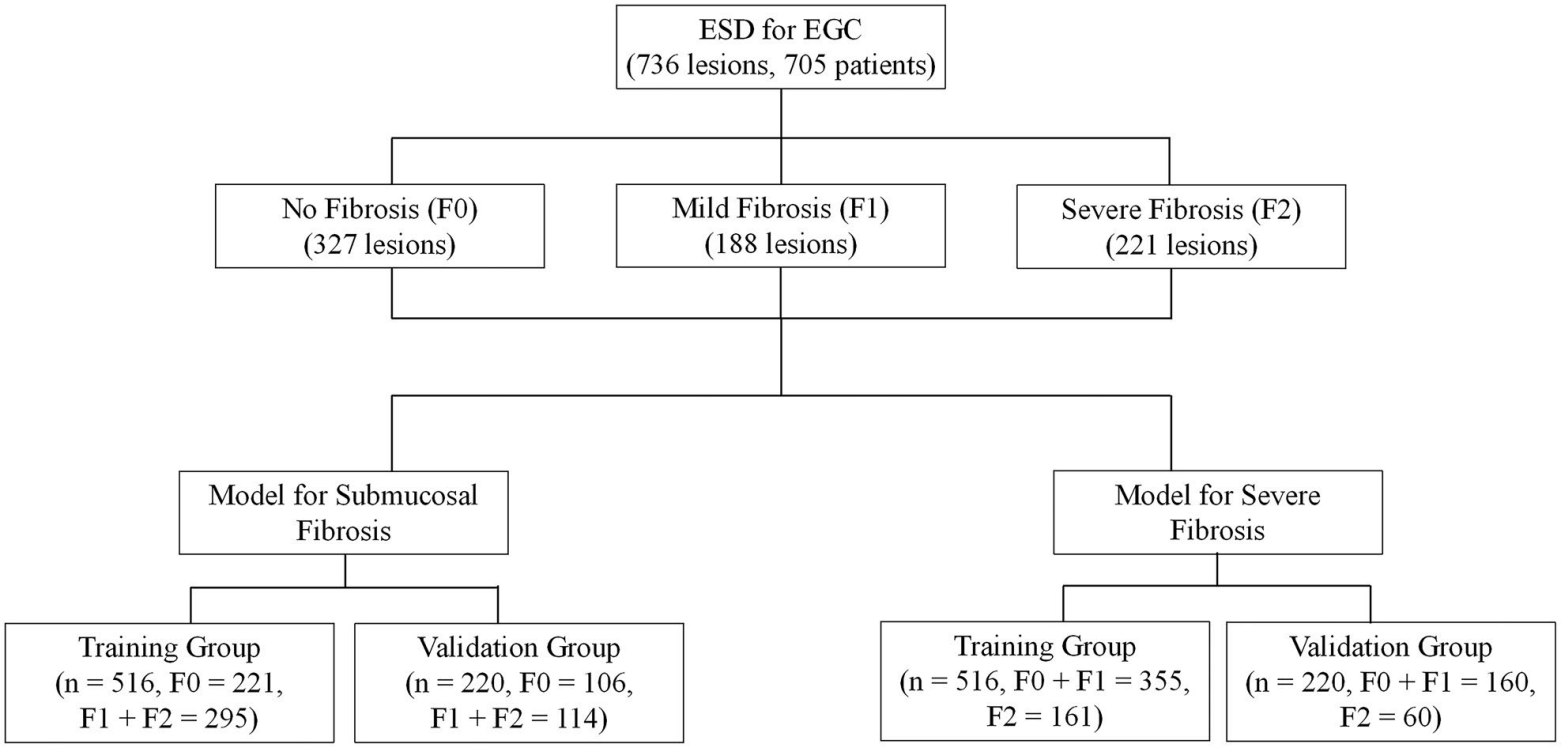
Study flow diagram. EGC, early gastric cancer; ESD, endoscopic submucosal dissection; F0, no fibrosis; F1, mild fibrosis; F2, severe fibrosis.

A total of 736 EGC cases were identified from our electronic medical record system, including 516 lesions in the training group and 220 lesions in the validation group (Figure 2). Baseline characteristics of the variables are displayed in Table 1. No significant differences in demographic and clinicopathological distribution were found between the training and validation groups, indicating the data of the training and validation sets were comparable.

**Table 1.** Basic characteristics of the training set and validation set.

Variables	Total (*n* = 736)	Training set (*n* = 516)	Validation set (*n* = 220)	*P*
Age, years, median [IQR]	65 [58, 71]	65 [59, 71]	64 [57, 71]	0.453
Male gender, n (%)	583 (79.2)	412 (79.8)	171 (77.7)	0.517
Size of tumour, n (%)				0.930
<30 mm	537 (73.0)	376 (72.9)	161 (73.2)	
≥ 30 mm	199 (27.0)	140 (27.1)	59 (26.8)	
Tumour location long axis, n (%)				0.916
Upper	333 (45.2)	236 (45.7)	97 (44.1)	
Middle	96 (13.0)	67 (13.0)	29 (13.2)	
Lower	307 (41.7)	213 (41.3)	94 (42.7)	
Tumour location short axis, n (%)				0.782
Lesser	372 (50.5)	256 (49.6)	116 (52.7)	
Greater	104 (14.1)	77 (14.9)	27 (12.3)	
Anterior	80 (10.9)	56 (10.9)	24 (10.9)	
Posterior	180 (24.5)	127 (24.6)	53 (24.1)	
Endoscopic findings, n (%)				
Atrophy	682 (92.7)	477 (92.4)	205 (93.2)	0.725
Ulceration	166 (22.6)	118 (22.9)	48 (21.8)	0.755
Depression	553 (75.1)	385 (74.6)	168 (76.4)	0.615
Spontaneous bleeding	95 (12.9)	65 (12.6)	30 (13.6)	0.700
Redness	708 (96.2)	494 (95.7)	214 (97.3)	0.319
White fur	210 (28.5)	146 (28.3)	64 (29.1)	0.827
Dyke-like elevated margins	44 (6.0)	28 (5.4)	16 (7.3)	0.333
Biopsy pathology, n (%)				0.606
LGIN	36 (4.9)	27 (5.2)	9 (4.1)	
HGIN	373 (50.7)	256 (49.6)	117 (53.2)	
Adenocarcinoma	327 (44.4)	233 (45.2)	94 (42.7)	
Time of ESD from initial biopsy, n (%)				0.629
≤21 d	425 (57.7)	295 (57.2)	130 (59.1)	
>21 d	311 (42.3)	221 (42.8)	90 (40.9)	
Biopsy times, n (%)				0.381
<2 times	626 (85.1)	435 (84.3)	191 (86.8)	
≥2 times	110 (14.9)	81 (15.7)	29 (13.2)	
Antithrombotic drug use, n (%)	48 (6.5)	35 (6.8)	13 (5.9)	0.660
Comorbidity				
CAD/AF, n (%)	106 (14.4)	79 (15.3)	27 (12.3)	0.283
Diabetes mellitus, n (%)	114 (15.5)	82 (15.9)	32 (14.5)	0.644
Hypertension, n (%)	253 (34.4)	183 (35.5)	70 (31.8)	0.340
Cirrhosis, n (%)	11 (1.5)	7 (1.4)	4 (1.8)	0.637
Degree of submucosal fibrosis, n (%)				0.181
F0	327 (44.4)	221 (42.8)	106 (48.2)	
F1/F2	409 (55.6)	295 (57.2)	114 (51.8)	

AF, atrial fibrillation; CAD, coronary artery disease; ESD, endoscopic submucosal dissection; HGIN, high-grade intraepithelial neoplasia; IQR, interquartile range; LGIN, low-grade intraepithelial neoplasia.

### 3.2. Risk factors for submucosal fibrosis in EGC

Univariate logistic analysis indicated that male gender, tumour location (long axis), tumour location (short axis), ulceration, spontaneous bleeding, and biopsy pathology were associated with submucosal fibrosis in EGC in the training group. Multivariate logistic analysis revealed that tumour location (long axis), tumour location (short axis), and ulceration were independent factors predicting the risk of submucosal fibrosis. Lesions in the middle (OR = 4.317, 95% CI = 2.342–7.957, *p* < 0.001) and upper (OR = 16.187, 95% CI = 9.599–27.296, *p* < 0.001) location were more prone to submucosal fibrosis than those in the lower third of stomach. Additionally, tumors located in the lesser curvature (OR = 2.463, 95% CI = 1.175–5.164, *p* = 0.017) and those with ulceration (OR = 2.594, 95% CI = 1.514–4.444, *p* = 0.001) were also significantly associated with submucosal fibrosis (Table 2).

**Table 2.** Univariate and multivariate analyses of factors associated with submucosal fibrosis in EGC.

Variables	OR (95%CI)	*P*	aOR (95%CI)	a*P*
Age	1.005 (0.985-1.026)	0.637		
Male gender	1.663 (1.079-2.563)	0.021	1.199 (0.703-2.046)	0.505
Size of tumour, mm				
<30	1			
≥30	1.440 (0.966-2.148)	0.074		
Tumour location: long axis				
Lower	1		1	
Upper	13.825 (8.742-21.862)	<.001	16.187 (9.599-27.296)	<.001
Middle	4.316 (2.423-7.689)	<.001	4.317 (2.342-7.957)	<.001
Tumour location: short axis				
Anterior	1		1	
Greater	1.897 (0.932-3.863)	0.077	1.995 (0.841-4.732)	0.117
Lesser	2.893 (1.577-5.308)	0.001	2.463 (1.175-5.164)	0.017
Posterior	3.941 (2.026-7.666)	<.001	1.461 (0.642-3.327)	0.367
Endoscopic findings				
Atrophy	0.822 (0.421,1.607)	0.567		
Ulceration	1.707 (1.109-2.627)	0.015	2.594 (1.514-4.444)	0.001
Depression	1.225 (0.823-1.826)	0.318		
Spontaneous bleeding	1.809 (1.035-3.162)	0.038	1.660 (0.849-3.247)	0.139
Redness	1.352 (0.575-3.179)	0.489		
White fur	1.459 (0.984-2.165)	0.060		
Dyke-like elevated margins	0.857 (0.399-1.840)	0.693		
Biopsy pathology				
Adenocarcinoma	1		1	
HGIN	0.824 (0.574-1.182)	0.293	0.990 (0.636-1.540)	0.963
LGIN	0.320 (0.138-0.744)	0.008	0.415 (0.148-1.166)	0.095
Time of ESD from initial biopsy >21 d	0.739 (0.520-1.052)	0.093		
Biopsy performed ≥2 times	1.176 (0.725-1.909)	0.511		
Antithrombotic drug use	0.999 (0.499-1.998)	0.997		
Comorbidity				
CAD/AF	0.932 (0.575-1.510)	0.774		
Diabetes mellitus	1.205 (0.744-1.953)	0.448		
Hypertension	1.205 (0.836-1.739)	0.318		
Cirrhosis	0.999 (0.221-4.509)	0.999		

AF, atrial fibrillation; CAD, coronary artery disease; EGC, early gastric cancer; ESD, endoscopic submucosal dissection; HGIN, high-grade intraepithelial neoplasia; IQR, interquartile range; LGIN, low-grade intraepithelial neoplasia.

### 3.3. Nomogram for submucosal fibrosis prediction

**Figure 3.**
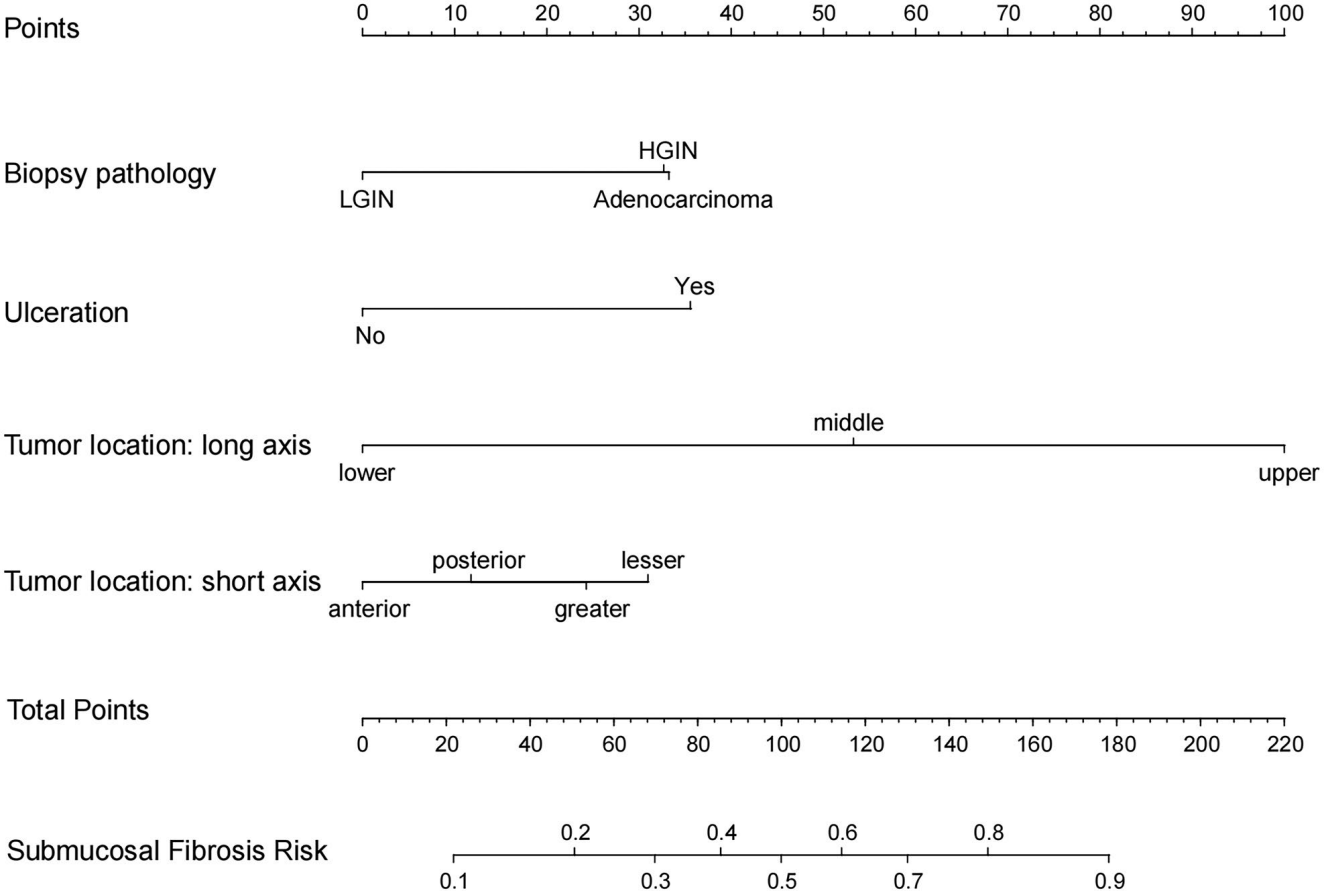
Nomogram for predicting the probability of submucosal fibrosis in early gastric cancer. HGIN, high-grade intraepithelial neoplasia; LGIN, low-grade intraepithelial neoplasia.

To visualize the model for predicting the probability of submucosal fibrosis in EGC patients, a nomogram was plotted based on the independent risk factors (long-axis location, short-axis location, and ulceration) derived from the multivariate logistic regression analysis in the training group (Figure 3). One additional variable (biopsy pathology) was also included, considering its clinical significance and corresponding OR value in the univariate analysis. As shown in the nomogram, tumour location (long axis) contributed the greatest significant influence on the fibrosis outcome. Each variable corresponded to a particular point. The overall score was calculated as the cumulative total of ratings attributed to the four constituent factors, and the corresponding risk represented the probability of a patient developing submucosal fibrosis.

**Figure 4.**
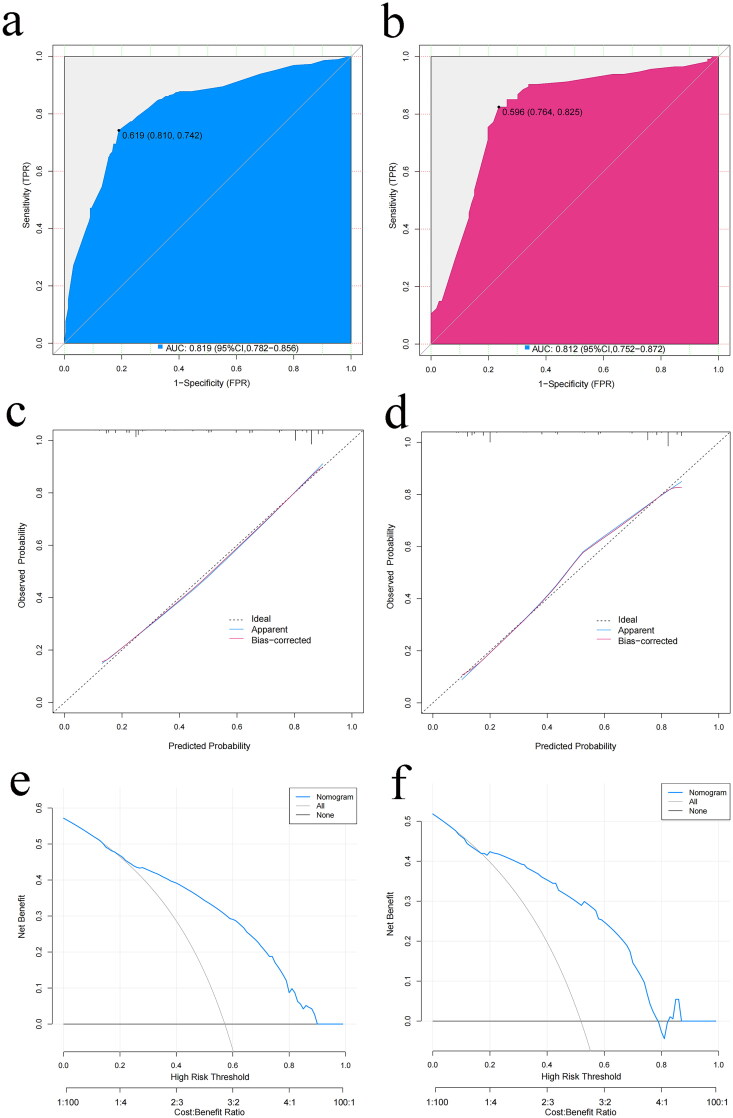
Receiver operating characteristic curves of the predictive model in the (a) training group and (b) validation group. Calibration curves of the predictive model in the (c) training group and (d) validation group. Decision curve analysis of the prediction model in the (e) training group and (f) validation group. AUC, area under the curve; FPR, false positive rate; TPR, true positive rate.

The ROC curve was plotted to test the effectiveness of the model. The model showed an excellent capability to distinguish the presence or absence of submucosal fibrosis, with AUC, specificity, and sensitivity values of 0.819, 0.810, and 0.742 and 0.812, 0.764, and 0.825 in the training and validation groups, respectively (Figure 4a and 4b).

Then, the performance of the model was evaluated using the calibration plots, and the results revealed that the model exhibited strong calibration ability regardless of the training group (Figure 4c) or the validation group (Figure 4d). Additionally, the H-L test showed that the *P-*value was 0.566 (χ^2^ = 6.73) and 0.864 (χ^2^ = 3.93) in the training and validation groups, respectively. These results demonstrated that the predictive model could estimate the risk of submucosal fibrosis and was highly congruent with the real risk.

DCA was performed to identify the net benefit of the model. It was found that the nomogram in the training group could provide benefits when the threshold probability was between 0.18 and 0.89 (Figure 4e). In the validation group, the curve indicated that patients with an 18–78% threshold probability would benefit from this prediction model for submucosal fibrosis (Figure 4f).

### 3.4. Predictive model of severe submucosal fibrosis in EGC

**Figure 5.**
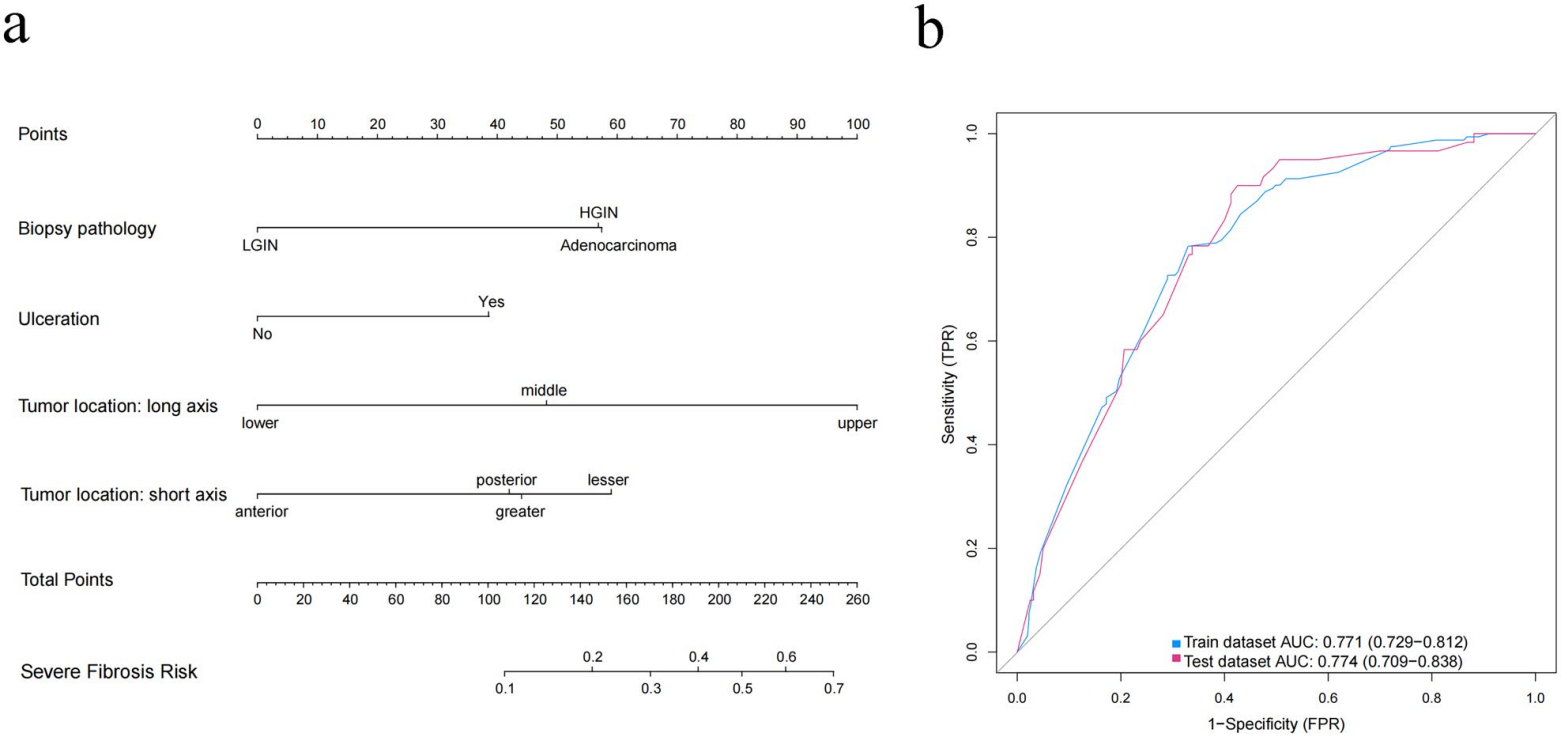
(a) Nomogram for predicting the probability of severe submucosal fibrosis in early gastric cancer. (b) Receiver operating characteristic curves of the predictive model in the training group and validation group. AUC, area under the curve; FPR, false positive rate; HGIN, high-grade intraepithelial neoplasia; LGIN, low-grade intraepithelial neoplasia; TPR, true positive rate.

We further attempted to conduct a nomogram to predict the probability of severe submucosal fibrosis in patients with EGC, employing a similar methodology. Based on multivariate regression analysis, four predictors (long-axis location, short-axis location, ulceration, and biopsy pathology) incorporated in the preceding nomogram for submucosal fibrosis were still applicable for the prediction of severe fibrosis (Table 3 and Figure 5a). Besides, a patient’s probability of F2 fibrosis required higher total points compared with submucosal fibrosis. The ROC curve showed high efficiency in the training group, with an AUC of 0.771, and the effectiveness was verified in the validation group, with an AUC of 0.774 (Figure 5b). H-L tests (Training set: *p* = 0.546; Validation set: *p* = 0.622) also revealed good agreement, indicating that the nomogram was reliable.

**Table 3.** Univariate and multivariate analyses of factors associated with severe submucosal fibrosis in EGC.

Variables	OR (95%CI)	*P*	aOR (95%CI)	a*P*
Age	0.999 (0.977-1.021)	0.911		
Male gender	1.462 (0.896-2.385)	0.128		
Size of tumour, mm				
<30	1			
≥30	1.214 (0.804-1.835)	0.357		
Tumour location: long axis				
Lower	1		1	
Upper	8.010 (4.880-13.146)	<.001	8.457 (4.946-14.459)	<.001
Middle	2.893 (1.455-5.751)	0.002	2.799 (1.373-5.706)	0.005
Tumour location: short axis				
Anterior	1		1	
Greater	2.542 (0.937-6.896)	0.067	2.561 (0.877-7.481)	0.086
Lesser	4.290 (1.770-10.399)	0.001	3.524 (1.376-9.027)	0.009
Posterior	5.411 (2.160-13.559)	0.000	2.451 (0.919-6.539)	0.073
Endoscopic findings				
Atrophy	0.795 (0.402-1.574)	0.511		
Ulceration	1.729 (1.128-2.651)	0.012	2.277 (1.383-3.751)	0.001
Depression	1.334 (0.858-2.075)	0.201		
Spontaneous bleeding	1.448 (0.845-2.481)	0.178		
Redness	1.569 (0.569-4.330)	0.384		
White fur	1.443 (0.963-2.161)	0.076		
Dyke-like elevated margins	1.047 (0.463-2.368)	0.912		
Biopsy pathology				
Adenocarcinoma	1		1	
HGIN	0.838 (0.573-1.225)	0.361	0.988 (0.646-1.513)	0.957
LGIN	0.239 (0.070-0.818)	0.023	0.294 (0.079-1.095)	0.068
Time of ESD from initial biopsy >21 d	1.002 (0.687-1.459)	0.993		
Biopsy performed ≥2 times	1.365 (0.832-2.240)	0.218		
Antithrombotic drug use	1.163 (0.563-2.398)	0.684		
Comorbidity				
CAD/AF	1.430 (0.869-2.354)	0.159		
Diabetes mellitus	1.421 (0.870-2.323)	0.161		
Hypertension	0.996 (0.675-1.470)	0.984		
Cirrhosis	0.881 (0.169-4.587)	0.880		

AF, atrial fibrillation; CAD, coronary artery disease; EGC, early gastric cancer; ESD, endoscopic submucosal dissection; HGIN, high-grade intraepithelial neoplasia; IQR, interquartile range; LGIN, low-grade intraepithelial neoplasia.

## 4. Discussion

This study demonstrated that the risk of submucosal fibrosis during gastric ESD was higher in EGCs with ulceration, in the upper third stomach and the lesser curvature. EGC diagnosed as low-grade intraepithelial neoplasia (LGIN) on EFB was relatively less prone to submucosal fibrosis. These variables were also related to severe fibrosis. Therefore, clinicians should be vigilant when identifying these factors. The probability of submucosal fibrosis is complex and cannot be accurately predicted by any single factor. Hence, we integrated these significant factors and established a prediction model.

To date, several studies exploring the influence of submucosal fibrosis on gastric ESD outcomes have roughly assessed factors associated with submucosal fibrosis [2,11]. These studies focused not only on pre-ESD predictive factors but also on post-ESD factors, such as the final pathology of the tumour. Our study examined more endoscopy-related variables (i.e. atrophy and dyke-like elevated margins), and specifically used pre-ESD factors to establish prediction models for both submucosal fibrosis and severe fibrosis.

Although our study enrolled patients diagnosed with EGC by post-ESD pathology, biopsy pathology was analyzed instead of post-ESD pathology to predict submucosal fibrosis during the ESD procedure. The results showed that the risk of submucosal fibrosis was lower in EGC diagnosed as LGIN on EFB but with no statistical significance in the multivariate analysis, perhaps due to unavoidable pathologic discrepancy between EFB and ESD. Previous reports strongly suggested that high-grade intraepithelial neoplasia diagnosed by EFB is highly predictive of the coexistence of invasive carcinoma compared with LGIN [17,18]. The depth of invasion has been proven to play an important role in predicting either submucosal fibrosis or severe fibrosis [12,19]. As the tumour invades, there is an increase in total collagen that is progressively thickened and linearized. The invasive front of the tumors particularly displayed an enhancement of aligned and stiffer collagen fibres [20]. Therefore, tumors with deep invasion are commonly accompanied by interstitial fibrosis [15].

For lesions with ulcer formation, submucosal adhesions are often found during the procedure [21]. Ulceration on the surface of a lesion is suggestive of deep tumour invasion. However, ulceration can still induce deep submucosal fibrosis, even in the absence of such invasion [22–24]. Deep ulceration prompts an increase in the production and deposition of collagen fibres, and continuous exposure to gastric contents induces severe inflammation and fibrosis, potentially leading to excessive scarring [25–30]. A critical event in the ulcer healing process is the transformation of fibroblasts into myofibroblasts, which express smooth muscle actin and create robust contractile forces [31–33]. Fibrosis causes regenerative mucosa to aggregate and contract, thereby covering the mucosal defect [34]. In our study, endoscopic ulceration was an independent factor for predicting the risk of both submucosal fibrosis and severe submucosal fibrosis during ESD, consistent with the finding of Higashimaya et al. [2]. Intriguingly, severe submucosal fibrosis was also reported as an independent predictor of delayed ESD-related artificial ulcer healing due to significant proper muscle layer damage caused by persistent electrocautery [35,36]. However, the relationship between ulceration and submucosal fibrosis lacked statistical significance in Jeong et al.’s multivariate regression analysis [11]. The specific role of ulceration in gastric fibrosis still requires further elucidation.

Submucosal fibrosis of varying degrees was more prevalent in the current study than previously reported, possibly due to a greater proportion of tumors in the upper/middle third stomach, particularly in the cardia [2,11]. Tumour location was another significant risk factor in our study. According to the nomograms, tumour location (long axis) had the largest impact on the risk of submucosal fibrosis and severe fibrosis. The closer the tumour was to the proximal portion of the stomach, the more severe the submucosal fibrosis was, aligning with findings from previous studies [2,11]. Regurgitation, a primary risk factor for cardia gastric adenocarcinoma, likely causes the observed submucosal fibrosis through recurrent injuries and chronic inflammation, which promote collagen production and deposition [12,25,37–40]. Another reason may be that submucosa-invasive EGCs are more frequently observed in the middle/upper third stomach, where early detection of EGC is challenging and EGC is more vulnerable to infiltration into the submucosa due to the thinner submucosa and gastric wall in the proximal stomach [23,41,42].

Lesser curvature, the most common site on the short-axis of tumour occurrence [43,44], showed increased susceptibility to submucosal fibrosis and severe fibrosis in EGC, consistent with the finding of Konuma et al. [45]. Nevertheless, the cause remains to be elucidated. The relatively constant position of gastric ulcers and scars on the lesser curvature may explain the association between fibrosis and the lesser curvature [45,46]. Ulcers on the lesser curvature also heal more slowly due to the severe kinetic strain caused by gastric motility [47,48]. Besides, chronic inflammation caused by *Helicobacter pylori* infection also advances along the lesser curvature [43]. Tumors located in the proximal stomach and along the lesser curvature are inherently associated with prolonged hemostasis times during ESD. The presence of submucosal fibrosis exacerbates this challenge by increasing perilesional vascularity, further complicating the haemostatic process [45]. Since tumour location is the most readily predictable factor for submucosal fibrosis, endoscopists should recognize and be prepared in advance.

The role of biopsy in detecting submucosal fibrosis during oesophageal and colorectal ESD has been widely explored [15,16,49,50], with delayed procedures beyond 21 days post-biopsy potentially causing ulceration, scarring, and subsequent fibrosis [50,51]. However, there is limited research on the association between preoperative biopsy and fibrosis in gastric lesions. Our findings revealed that the interval between initial biopsy and gastric ESD and biopsy times had little relationship with submucosal fibrosis in EGC patients. Jeong et al. also reported that the time from diagnostic biopsy to gastric ESD did not differ between the non-fibrosis and fibrosis groups [11]. Zhuang et al. suggested that standard biopsy forceps result in minor acute mucosal defects quickly repaired by epithelial cell proliferation, unlike the chronic inflammation driving pathogenic fibrosis [15,52]. Therefore, endoscopists may not need to immediately perform ESD within 21 days after a biopsy to prevent submucosal fibrosis.

The significance of this study lies in its successful establishment of a model to predict submucosal fibrosis before ESD, which is the first of its kind. ROC and DCA curves demonstrate the great utility and excellent diagnostic effectiveness of the model. In addition, clinicians can predict submucosal fibrosis and severe submucosal fibrosis simultaneously using only four readily available clinical factors. A higher total score from these factors correlates with increased fibrosis severity. Our models allow for timely pre-ESD risk evaluation and heightened preparedness for potential complications. Especially, accurate pre-ESD prediction of severe fibrosis ensures experienced specialist involvement and optimal incision approach [16,53]. It also facilitates the use of effective traction for better submucosal layer visualization and high-viscosity injection fluids to enhance the submucosal lifting effect [54–57]. The strengths of our study encompass a large sample size, a broad time span, and an exhaustive examination of potential risk factors.

Nonetheless, this study has several limitations. First, this is a retrospective, single-center study, and the established predictive models were not externally validated. Second, we evaluated the grade of fibrosis based on endoscopic findings rather than histopathology, in line with many studies [12,13,15,49]. While endoscopic assessment relies on the endoscopist’s experience or judgment, histological assessment may offer greater objectivity. However, high concordance between endoscopic and histologic estimates of submucosal fibrosis severity has been confirmed [2,11,50]. Besides, our data showed a good interevaluator agreement of submucosal fibrosis (kappa = 0.887). Additionally, in some cases, histological fibrosis differs from endoscopic fibrosis in the submucosal layer since the depth of dissection or thermal injury by electrocoagulation can influence histological fibrosis [58]. More importantly, submucosal fibrosis during the ESD procedure has more clinical importance than histological fibrosis [16].

In conclusion, an accurate and effective nomogram was constructed based on several clinicopathological features to predict submucosal fibrosis in patients with EGC undergoing ESD. The predictive model facilitates timely assessment of risk and treatment decision-making.

## Data Availability

The data that support the findings of this study are available on reasonable request from the corresponding author.
